# Muscle MRI in immune-mediated necrotizing myopathy (IMNM): implications for clinical management and treatment strategies

**DOI:** 10.1007/s00415-022-11447-7

**Published:** 2022-11-03

**Authors:** Laura Fionda, Antonio Lauletta, Luca Leonardi, Jorge Alonso Perez, Stefania Morino, Gioia Merlonghi, Girolamo Alfieri, Rocco Costanzo, Laura Tufano, Fiammetta Vanoli, Elena Rossini, Eduard Gallardo Vigo, Tommaso Tartaglione, Marco Salvetti, Giovanni Antonini, Jordi Diaz-Manera, Matteo Garibaldi

**Affiliations:** 1 Neuromuscular and Rare Disease Centre, Department of Neuroscience, Mental Health and Sensory Organs (NESMOS), SAPIENZA University of Rome, Sant’Andrea Hospital, 00189 Rome, Italy; 2 Neurology Department, Neuromuscular Disorders Unit, Universitat Autònoma de Barcelona, Hospital de la Santa Creu I Sant Pau, 08041 Barcelona, Spain; 3 IRCCS Istituto Neurologico Carlo Besta, Milan, Italy; 4 Department of Radiology, Istituto Dermopatico dell’Immacolata, IRCCS, 00167 Rome, Italy; 5 John Walton Muscular Dystrophy Research Centre, Translational and Clinical Research Institute, Newcastle University, Newcastle Hospitals NHS Foundation Trust, Newcastle Upon Tyne, NE1 3BZ UK; 6 Centro de Investigación Biomédica en Red en Enfermedades Raras (CIBERER), 08041 Barcelona, Spain

**Keywords:** Immune mediated necrotizing myopathy (IMNM), Whole body muscle MRI, Inflammatory myopathies, Immunomodulating therapy, Follow-up study

## Abstract

**Objectives:**

Immune-mediated necrotizing myopathy (IMNM) is the most severe idiopathic inflammatory myopathy (IIM) and early aggressive poly-immunotherapy is often required to reduce long-term disability. The aim of this study is to investigate muscle MRI in IMNM as outcome measure for disease activity, severity, progression, response to treatment, and to better characterize the pattern of muscle involvement.

**Methods:**

This is a retrospective, observational, cross-sectional, and longitudinal study including 22 IMNM patients, divided into three groups based on timing of first MRI and if performed before or under treatment. T1 score and percentage of STIR positive muscles (STIR%) were considered and analyzed also in relation to demographic, clinical and laboratory characteristics.

**Results:**

STIR% was higher in untreated patients and in those who performed MRI earlier (*p* = 0.001). Pelvic girdle and thighs were in general more affected than legs. T1 score was higher in patients with MRI performed later in disease course (*p* = 0.004) with a prevalent involvement of the lumbar paraspinal muscles, *gluteus medius* and *minimus*, *adductor magnus* and hamstrings. 22% of STIR positive muscles showed fat replacement progression at second MRI. Higher STIR% at baseline correlated with higher risk of fat replacement at follow-up (*p* = 0.003); higher T1 score correlated with clinical disability at follow-up, with late treatment start and delayed treatment with IVIG (*p* = 0.03).

**Interpretation:**

Muscle MRI is a sensitive biomarker for monitoring disease activity and therapy response, especially when performed early in disease course and before treatment start, and could represent a supportive outcome measure and early prognostic index in IMNM.

## Introduction

Immune-mediated necrotizing myopathy (IMNM) is a specific nosological entity of idiopathic inflammatory myopathy (IIM) characterized by rapidly progressive muscular weakness with marked increase of serum creatine kinase (CK) and prominent myofiber necrosis and regeneration with mild or absent inflammatory infiltrates at muscle biopsy [1, 2]. Two different antibodies have been associated to IMNM: the anti-signal recognition particle (SRP) and the anti-hydroxy-3-methylglutaryl-CoA reductase (HMGCR), that account for about two-thirds of IMNM patients [3]. IMNM, may be triggered by statin exposure in about two-thirds of HMGCR positive cases [4]. Conversely, anti-SRP patients usually show severe clinical presentation, with higher frequency of cardiac involvement, extramuscular manifestations, and malignancy [5]. A third group of seronegative IMNM (anti-SRP and anti-HMGCR negative) represents about one-third of patients and shares distinctive features, including female predominance, higher frequency of connective tissue disorders and malignancy [6]. Treatment strategies include conventional first-line treatments (corticosteroids), but frequently require second- (and/or immunosuppressive agents) or eventually third-line (IVIG or Rituximab) immunotherapy to achieve the best clinical outcome [7]. IMNM represents the most severe form of IIM and early aggressive poly-immunotherapy is often required to reduce the long-term residual disability [8]. This could be easily detected by muscle magnetic resonance (MRI), which represents the gold standard technique for muscle imaging study in muscle diseases [9]. Despite its usefulness, it is largely established for inherited myopathies to recognize the pattern and severity of fat replacement by T1-sequences and muscle edema/inflammation by T2-Short-tau-inversion-recovery (STIR)-sequences [10], its application to IIM, and to IMNM in particular, is still not of customary use. Muscle MRI could be helpful to assess disease activity and severity in IMNM, other than to identify the target site for biopsy [11]. Some studies investigated the role of muscle MRI in IIM showing that it could play a role for monitoring the disease progression and response to therapy [12, 13]. Concerning IMNM, muscle MRI showed a higher proportion of thigh muscle oedema, atrophy and fat replacement compared to other IIMs with more severe muscle involvement in SRP than HMGCR positive patients [14–16].

Nevertheless, the pattern of muscle involvement by MRI and its longitudinal use for monitoring disease progression and treatment response in IMNM is still warranted.

In this framework, we investigated the use of muscle MRI to establish its usefulness as prognostic tool for disease severity, progression, and treatment response and to better characterize the pattern of the muscle involvement in IMNM.

## Materials and methods

### Patients

This is a retrospective, observational, cross-sectional, and longitudinal study including IMNM patients followed at Sant’Andrea Hospital of Sapienza University of Rome and at Hospital de la Santa Creu i Sant Pau of UAB University of Barcelona. Patients’ informed consent to MRI, which includes a statement about image preservation for research, was obtained from all participants prior to performing MRI, in compliance with ethical standards of local ethical committees, the Helsinki Declaration, and the Good Clinical Practice.

All patients received a diagnosis of IMNM accordingly to the 224th European Neuromuscular Centre (ENMC) international workshop on IMNM [3], in particular muscle biopsy required the presence of necrosis and regeneration at different stages, poor macrophagic-prevalent inflammatory infiltrates, variable and faint expression of MHC-I and c5b-9 complement deposition on sarcolemma with supportive detection of antibodies against SRP or HMGCR. All patients performed at least one MRI study including STIR and T1 sequences of between January 1, 2014 at the date of January 1, 2020 for diagnostic and clinical purposes. In the participating centers, the diagnostic workup in suspected IIM includes a full clinical evaluation, muscle MRI study, muscle biopsy and laboratory analysis with CK and antibody testing. All patients underwent a full screening for malignancy during the diagnostic workup.

All patients underwent clinical examination at baseline and during the clinical follow-up. Neurological examination included a revised version of the MRC-sum score for proximal muscles (MRC-60) obtained from the following muscle groups of each side: arm abductors, elbow flexors, elbow extensors, hip flexors, knee flexors and knee extensors. Since MRC sum score provide a 5-point scale of evaluation, the maximum score allowed by summing the strength of each group of muscles aforementioned is 60, so we called this parameter MRC-60. Only evaluations performed in proximity of the MRI studies (± 1 month) were considered for statistical analysis. A comprehensive clinical evaluation including muscle strength evaluation of facial, bulbar, axial and upper and lower limb impairment scored by MRC scale, were used to establish the overall disability for clinical outcome purpose: asymptomatic (no muscular weakness), mild (MRC-60 score: 41–60, mild axial weakness, without facial, bulbar, respiratory or distal muscle weakness, rise from the floor without aid), moderate (MRC-60 score: 21–40, moderate axial weakness, with minimal facial, bulbar, respiratory or distal muscle weakness, rise from the floor with aid) or severe (MRC-60 score: 0–20, moderate to severe axial, facial, bulbar, respiratory or distal muscle weakness, unable to rise from the floor). Disease remission was considered for patients with normal CK level and stationary best clinical outcome for at least 3 months without or tapering immunotherapy, regardless of residual clinical impairment. In patients with follow-up MRI study, STIR% score improvement > 90% at last MRI or STIR negativity at first MRI performed after 6 months from symptom onset (LATE group, see below) was also considered a supportive criterion.

For statistical analysis patients were divided into two major groups: (1) patients with MRI performed before 6 months from symptoms onset regardless of treatment (EARLY group) and (2) patients with MRI performed after 6 months from symptoms onset, who were all under treatment (LATE group). For supplementary analysis the EARLY group was also divided into patients who performed the first MRI before treatment (EARLY-BT subgroup) and patients who performed the first MRI under treatment (EARLY-UT).

### Laboratory analysis

All patients performed a full laboratory analysis, before starting treatment, including blood cell count, hepatic and renal function, thyroid hormones, lactate dehydrogenase (LDH), and serum creatine kinase (CK).

Patients' sera were tested for the presence of all myositis-specific autoantibodies (MSA) and myositis-associated autoantibodies (MAA) with a line blot test kit (Euroimmun, Lubeck, Germany) including 16 different antigens (Mi-2alfa, Mi-2beta, TIF1gamma, MDA5, NXP2, SAE1, Ku, PM-7, Scl100, PM-Scl75, Jo-1, SRP, PL-7, PL-12, EJ, OJ, Ro-52) [17]. Anti-HMGCR antibodies were studied using ELISA (Inova Diagnostics, San Diego, CA, USA) following the manufacturer instructions [18].

Serum CK activity was analyzed at diagnosis, at first MRI study (± 1 month) and last clinical follow-up for all but one of the patients. Analysis performed in proximity of the MRI studies (± 1 month) were considered for statistical analysis.

### Morphological study

Open muscle biopsy for diagnostic confirmation of IMNM was performed in all patients. Proximal upper or lower limb muscle was selected accordingly to clinical evaluation of muscle weakness or evidence of STIR positive signal at muscle MRI. In 13 patients, muscle MRI was performed before or within 1 month from muscle biopsy. Internal protocols were followed [19] accordingly to international standards of muscle biopsy [20] for conventional histological, histochemical and immunohistochemical techniques.

Open muscle biopsies were obtained from deltoid or quadriceps muscles in all patients. Conventional histological and histochemical techniques, 8–10 μm thick cryostat sections were stained with hematoxylin and eosin (HE), modified Gomori trichrome (GT), periodic acid Schiff technique (PAS), Oil red O, reduced nicotinamide adenine dinucleotide dehydrogenase-tetrazolium reductase (NADH-TR), succinic dehydrogenase (SDH) and cytochrome c oxidase (COX). The immunohistochemical (IHC) study using antibodies against CD3, CD4, CD8, CD31, CD56, CD68, HLA-ABC, HLA-DR and c5-b9 were matched with negative control slides and visualized using immunoperoxidase techniques.

Muscle biopsy was considered diagnostic for IMNM in presence of prominent myofiber necrosis and regeneration in different stages, mild or absent inflammatory cells, faint or absent sarcolemmal MHC-I positivity and c5b-9 complement deposition on sarcolemma in accordance with the international standards [21–23].

### Muscle MRI studies

All muscle MRIs were obtained using a 1.5-T MRI following previously described protocols [24] in accordance with the international consensus recommendations [25]. A total of 39 muscles of lower body (LB) including pelvic girdle and lower limb muscles and 18 muscles of upper body (UB) including scapular girdle and arms were studied from each side, analyzing T1 Turbo spin echo (T1-TSE) and T2-Short tau inversion recovery (T2-STIR) sequences. T1 and STIR sequences of scapular girdle or arms were not available for 3 and 7 patients, respectively. STIR sequences were not available for two patients at the first MRI acquisition.

Fat replacement was evaluated on T1 sequences using a 5-point scale (0–4) according to Fisher classification [26], while edema/inflammation on STIR sequences using a 2-point scale (0: negative, 1: positive).

STIR sequences were used to evaluate the presence of muscle inflammation and were considered as “positive” when an abnormally increased signal in the intra-muscular tissue could be detected, compared with the unaffected surrounding muscles. We calculated the percentage of STIR-positivity as a fraction of STIR-positive muscles over the total number of muscles evaluated (STIR% = total no STIR-positive muscles/total muscles evaluated*100) separately in UB and LB (STIR%UB and STIR%LB).

The overall burden of fat replacement was calculated for LB as a sum of total values of T1 score for each muscle per patient (T1-LB score) with a range between 0 (no fat replacement in any muscle) and 312 (complete replacement in all LB muscles) and it is also expressed as percentage of replacement.

For longitudinal evaluation of MRI changes, we evaluated the change over time as ΔSTIR and ΔT1 scores, between the first and last MRI available for each patient.

Two independent Neurologists with experience in MRI analysis (MG, LF) blinded to demographic and clinical features analyzed all MRIs. In muscles with different T1 and STIR scoring, observers reviewed the muscles together to agree the final score.

### Statistical analysis

Demographic, clinical and radiological features, including age at MRI, age at symptoms onset, disease duration, treatment start, delay and response, and time elapsed between symptoms onset, therapy start and MRI study, were collected retrospectively for each patient. For patients with more than one MRI study, only the first and last MRI was considered.

MRC scores and laboratory analysis (serum CK) were considered at the moment of the first MRIs (± 1 month) and at follow-up end. For subgroup analysis correlation and longitudinal studies, we considered only the scores acquired for the LB (STIR%LB and T1-LB and related Δ scores) because of lacking data for UB in some patients. We refer as “baseline” the data at the time of first MRI, and “follow-up” data at last MRI.

Cohen's kappa coefficient (κ) was calculated to assess the inter-rater agreement. Continuous variables were expressed as median, range and inter quantile range (IQR). Chi-square test was used for comparison of categorical variables. We identified that none of the variables analyzed was normally distributed using Kolmogorov–Smirnov and therefore we used non-parametric statistical studies. Mann–Whitney *U* test was used to identify whether differences observed between two groups were significant. Kruskal–Wallis with Dunn’s multiple comparison test was used for comparison of more than two groups (e.g., age of onset comparison between seronegative/HMGCR/SRP patients). Wilcoxon signed rank test was used to determine whether differences observed in continuous variables at two time points (baseline and follow-up) were statistically significant (e.g., T1-LB score at baseline and at the end of follow-up period). Spearman rank-order test was run to assess if correlations between variables were statistically significant. Correlation coefficients are expressed as *r* and considered strong correlation if higher than 0.8 and good if higher than 0.6. When multiple comparisons were performed, we applied a pos-hoc Bonferroni correction. Two-sided *p* values were calculated for all analyses; values of < 0.05 were considered significant.

All these analyses, as well as the graphics development, were performed using JASP Statistics 0.16 (IBM, Armonk, New York, USA) and GraphPad Prism 8.2.1. Hierarchical analysis, using mean fat replacement as the value analyzed, and graphical representation as a heatmap was performed using R software, V.4.0.3.

## Results

### Patients

Twenty-two patients (6 males, 16 females), aged between 23 and 83 years (mean 59.52 ± 17.35) were included in the study. Seven patients had anti-HMGCR antibodies, 8 anti-SRP antibodies and 7 were seronegative. There were no differences in median age at onset and gender prevalence in the different serotype groups. Muscle biopsies were performed on deltoid (15 patients), quadriceps (6 patients), biceps femoris (1 patient). Total body TC scan did not detect presence of cancer in any patients. Median disease duration at first MRI study was 4 months (IQR 27, range 0–330, SD 82.2).

Fifteen patients performed the first MRI before 6 months from symptom onset (EARLY group; median of 3 ± 1.84 months, range 0–6): of these, eleven patients were untreated at first MRI (EARLY-BT subgroup; median time of MRI from symptom onset: 2 months, range 1–6) and four were under treatment (EARLY-UT subgroup; all patients with corticosteroids from 1 month; median time of MRI from symptom onset 4 months, range 3–5). Seven patients performed MRI later than 6 months from symptom onset and all these patients were under treatment from a variable period (LATE group; median time of MRI from symptom onset: 66 ± 114 months, range 15–330).

Fourteen patients underwent more than one MRI, with a median follow-up duration of 16.5 months (IQR 15.5, range 4–30) between the first and the last MRI. Of these, eight patients belonging to the EARLY group (six patients EARLY-BT and two patients EARLY-UT group) while six patients to the LATE group.

All patients received immunotherapy during the disease course. Median time from symptoms onset to treatment start was 3 months (range 0–324). All but one patient received prednisone (up to 1 mg/kg/die) as first-line therapy. One patient received IVIG as first-line treatment with disease remission and mild residual disability. Four patients only received steroids obtaining disease remission in two, improvement in one and disease stabilization in one. Seventeen patients received a second-line therapy, with immunosuppressant agents (methotrexate *n* = 11; azathioprine *n* = 2; mycophenolate mofetil *n* = 1) or intravenous immunoglobulin (IVIg *n* = 3). Eleven patients also needed a third-line therapy to achieve the best clinical outcome with IVIg in six patients or a different immunosuppressant in five (cyclosporine A *n* = 2; rituximab *n* = 1; azathioprine *n* = 1; mycophenolate mofetil *n* = 1).

MRC-60 evaluation at baseline showed a median value of 48.5 (range 40–54, IQR 7), while at follow-up end it was 56.5 (range 48–60, IQR 6) (*p* < 0.0001).

CK values dropped from a mean of 6000 U/L (range 39–10,455, IQR 7792.5) at baseline to a mean of 154 U/L (range 37–887, IQR 202.5) at last visit (*p* < 0.0001).

At follow-up end, the best clinical outcome consisted of disease remission in 16 patients (8 asymptomatic and 8 with residual disability). Four patients improved without disease remission while two patients only achieved a disease stabilization without improvement. Of symptomatic patients, 11 had variable mild proximal weakness (with associated axial weakness in three, very mild in two, mild-moderate in two), and one patient had moderate proximal weakness of upper and lower limbs (MRC score 20–40). All patients still need therapy to maintain the best clinical outcome.

Major clinical and MRI characteristics of each patient are summarized in Table 1.

**Table 1:** Clinical and MRI features

Treatment	Group	FU MRI	Pt	Sex	MSA	Age (ys)	Timing MRI from DO/btw MRIs (mo)	STIR%LB first/second	ΔSTIR	T1-LB first/second	ΔT1	MRC BS/FU	CK onset/first MRI/FU end (UI/L)	No. of treatments	IVIG/delay (mo)	Disease outcome	Clinical outcome
*Untreated*																	
	Early-BT																
		Yes	P1	F	HMGCR	39	2/7	26/0	− 100%	4/4	0%	52/60	7400/7400/78	3	Y/6	R	A
			P2	F	HMGCR	34	1/10	46/4	− 92%	0/2	+ 0.6%	54/60	8800/8800/79	3	Y/5	R	A
			P3	F	HMGCR	74	6/9	49/0	− 100%	40/46	+ 1.9%	44/53	10,500/10,500/800	1	Y/6	R	Mild prox weakness
			P4	F	SRP	57	4/16	51/21	− 60%	15/110	+ 30.4%	40/56	6000/6000/250	3	Y/15	I	Mild prox weakness
			P5	F	SRP	78	1/4	37/0	− 100%	18/18	0%	43/57	8000/8000/80	3	Y/5	R	Very mild prox weakness
			P6	F	–	45	5/17	32/3	− 92%	11/15	+ 1.3%	50/60	3250/3250/150	2	N	R	A
		No	P7	M	HMGCR	61	3	52		31		42/50	25,000/25,000/2000	1	N	I	Mild prox weakness
			P8	F	SRP	83	2	n.a		30		48/58	3000/3000/95	2	N	R	Very mild prox weakness
			P9	M	–	65	0	27		29		54/60	n.a./n.a./290	1	N	R	A
			P10	F	–	84	4	58		102		26/34	3000/3000/96	2	N	I	Mod prox-dist-ax weakness
			P11	F	–	22	0	8		0		48/60	30,000/30,000/450	1	N	R	A
*Treated*																	
	Early-WT																
		Yes	P12	M	HMGCR	69	3/23	15/0	− 100%	50/50	0%	54/60	8200/8200/62	2	N	R	A
			P13	M	SRP	62	4/22	40/27	− 32%	31/54	+ 7.4%	49/60	7700/7700/250	2	Y/5–9	R	Axial weakness
		No	P14	F	SRP	66	5	21		36		43/60	4000/400/150	3	Y/5	R	A
			P15	F	–	83	3	14		62		48/52	2100/2100/25	1	N	I	Mild prox weakness
	Late																
		Yes	P16	M	HMGCR	76	53/30	4/1	− 66%	54/56	+ 0.6%	49/58	5300/410/270	3	Y/7	R	Mild prox weakness
			P17	F	HMGCR	30	330/15	0/0	–	162/162	0%	46/46	n.a./205/80	2	N	R	Mild prox weakness
			P18	F	SRP	62	66/27	2/4	+ 100%	62/62	0%	50/57	3600/170/150	3	N	R	Mild prox weakness
			P19	F	SRP	49	15/6	40/42	+ 6.5%	45/62	+ 5.4%	54/54	7300/5000/300	3	N	S	Mild prox weakness
			P20	F	SRP	53	181/210	0/0	0%	178/180	0%	48/48	3500/n.a./37	3	Y/96	R	Mild-mod prox weakness
			P21	F	–	39	164/17	23/23	0%	116/116	0%	48/48	n.a./n.a./n.a	3	N	S	Mild-mod prox-ax weakness
		No	P22	M	M	–	58	9		14		52/60	1200/n.a./700	3	Y/9	R	A

*FU* follow-up; *DO* disease onset; *btw* between; *BS* baseline; *Y* yes; *N* no; *R* remission; *I* improvement; *S* stabilization/refractory; *A* asymptomatic

### Cross-sectional MRI analysis

Considering the first MRI studies in all patients, the percentage of STIR positive muscles (%STIR) was similar in UB (STIR%UB: 26%, range 0–57%) and LB (STIR%LB: 22%; range 0–55%).

**Fig. 1:**
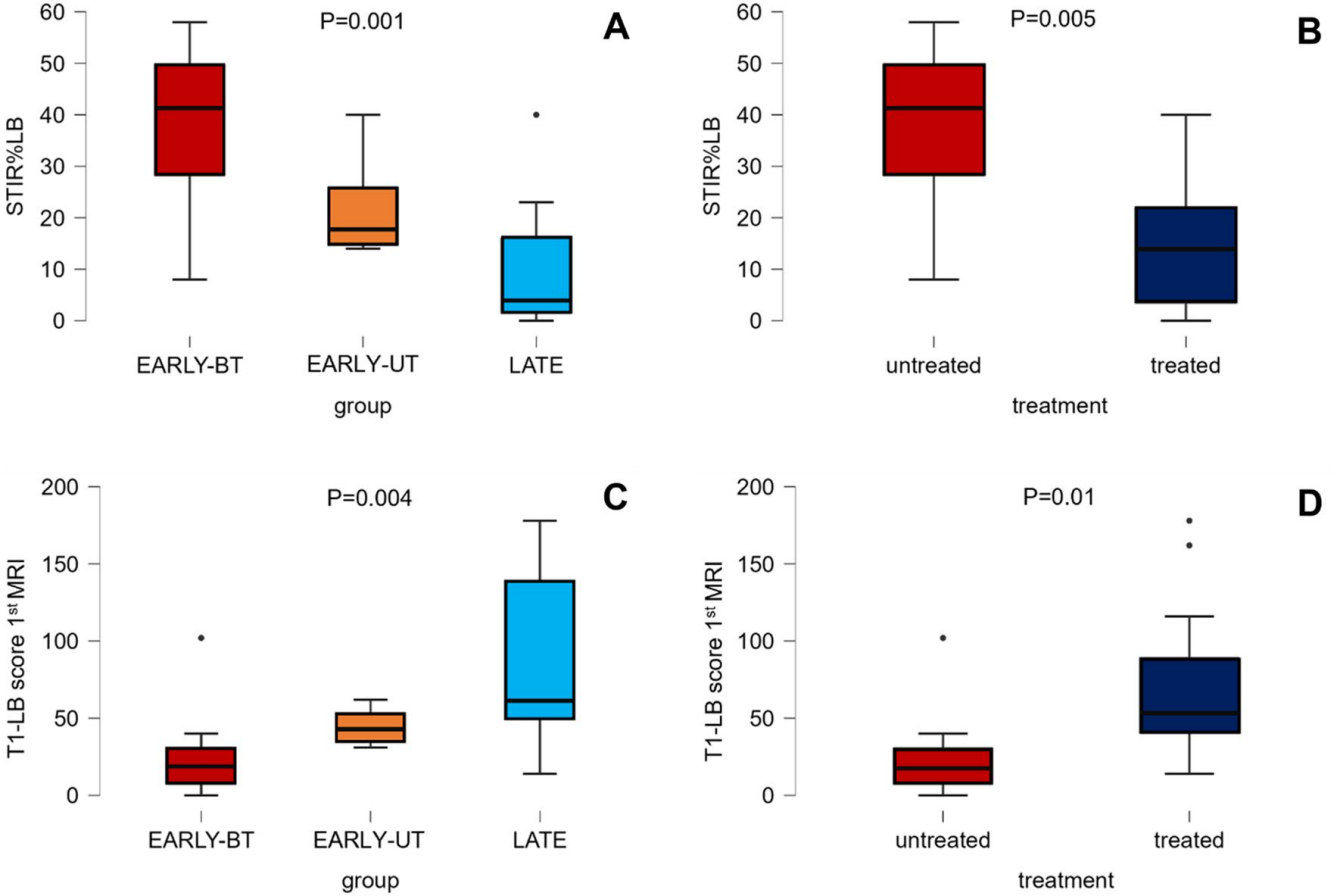
Median T1-LB and STIR%LB scores in EARLY/LATE and treated/untreated patients. Muscle MRI is more informative when performed early in disease course and before treatment, showing higher inflammation (higher STIR%LB score) in patients untreated (**B**) and with shorter disease duration (**A**), when muscles are still not replaced by fat (T1-LB score) fat with respect to patients with longer disease course (**C**) and under treatment (**D**)

The median score of STIR%LB positivity was 38% in EARLY-BT group (range 8–57%), 22% in EARLY-UT group (range 14–40%) and 11% in LATE group (range 0–40%) (*p* = 0.001). Considering treatment, the median score of STIR%LB positivity in treated patients was 15% (range 0–40%) versus the 38% of untreated patients (corresponding to the EARLY-BT group) (*p* < 0.005) (Fig. 1A, B).

**Fig. 2:**
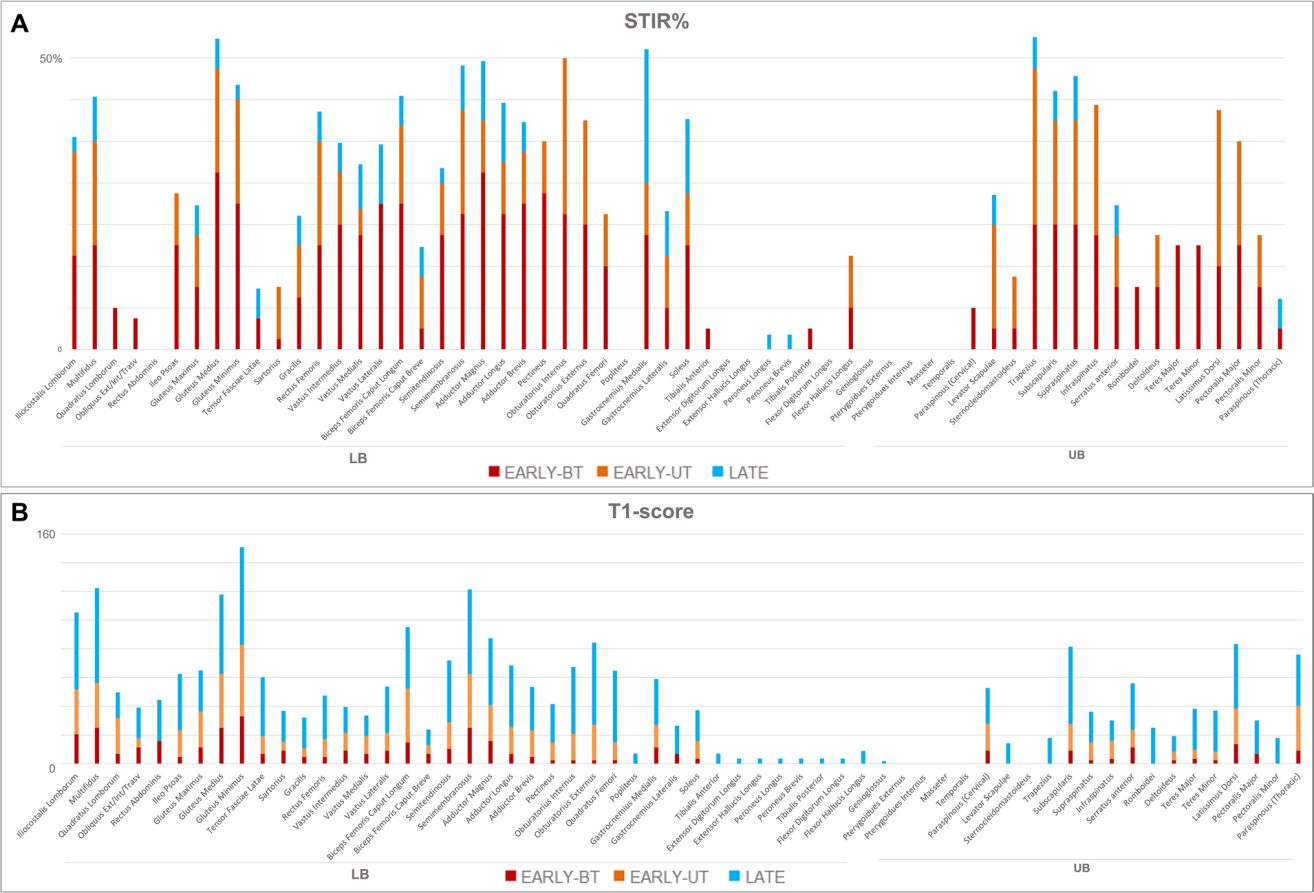
Single muscle STIR% and T1 score in upper and lower body. In LB STIR positivity is higher in pelvis and thighs than legs, with *adductor magnus*, *gluteus medius* and *obturatorius internus* and *gastrocnemius medialis* as the most frequently STIR positive muscles*,* followed by *gluteus minimus*, *biceps femoris caput longum*, *semimembranosus* and adductors (**A**, left). In the UB, *trapezius* and *supraspinatus* show the higher STIR positivity, followed by *subscapularis* and *infraspinatus* (**A**, right). Similarly, fat replacement occurs earlier in lumbar paraspinal muscles, *gluteus medius* and *minimus*, *adductor magnus* and hamstrings (**B**, left) in LB, and in subscapularis, latissimus dorsi and paraspinous muscles in UB (**B**, right). Each column represents the sum of scores obtained for each muscle in the totality of patients, subdivided in the three groups EARLY-BT (red), EARLY-UT (yellow) and LATE (blue)

In the LB, pelvic girdle and thighs were in general more affected than legs (32% and 34% versus 12% respectively). The most frequently STIR positive muscles were *adductor magnus* (57%), *gluteus medius* (55%) and *obturatorius internus* (50%) in LB, followed by *gluteus minimus*, *biceps femoris caput longum*, *semimembranosus, adductor longus* and *brevis*, *gastrocnemius medialis* (range of STIR positivity 45–48%) *Gastrocnemius medialis* was the most frequently affected STIR positive muscle in legs (52%). In the UB, *trapezius* and *supraspinatus* were the most affected muscles (55% and 50% respectively), followed by *subscapularis* (47%) and *infraspinatus* (42%) (Fig. 2A).

No differences of STIR%LB score were observed among the three serological subgroups (SRP: 29%; HMGCR: 26%; seronegative: 26%). However, a slight difference in the pattern of STIR positive muscles was observed. Lumbar, *gluteus minimus* and hamstring muscles were more commonly involved in SRP patients; *gluteus medius* and pelvic muscles in HMGCR-positive patients, while quadriceps and hamstring muscles were more frequently detected in seronegative patients.

T1 sequence analysis revealed a median T1-LB score of 33.5 (range 0–178). At first MRI, patients of EARLY-BT group had a median T1-LB score of 18 (average 28.4; 8% of replacement), while EARLY-UT group had a median T1-LB score of 43 (14% of replacement) and patients of LATE group had a median T1-LB score of 62 (29% of replacement) (*p* = 0.004). Considering treatment, the median T1-LB score in treated patients was 58 (range 31–178; average 77.8; 24% of replacement) versus the 18 of untreated patients (corresponding to the EARLY-BT group) (*p* = 0.01) (Fig. 1C, D).

Muscles with higher degree of fat replacement in LB were the lumbar paraspinal muscles, *gluteus medius* and *minimus*, *adductor magnus* and hamstrings, especially the *biceps femori caput longum* and the *semimembranosus* (Fig. 2B). In UB *subscapularis*, *latissimus dorsi* and *paraspinous* were the most affected muscles. No statistical differences were observed between the three serological subgroups in terms of T1-LB score and distribution of fat replacement.

### Longitudinal MRI analysis

A follow-up MRI study was performed in 14 patients with a median delay of 16.5 months from the first MRI (range 4–30, IQR 15.5). The average follow-up distance was 11 months in the EARLY-BT subgroup (*n* = 6), 23 months in the EARLY-UT subgroup (*n* = 2) and 21 months in the LATE group (*n* = 6).

**Fig. 3:**
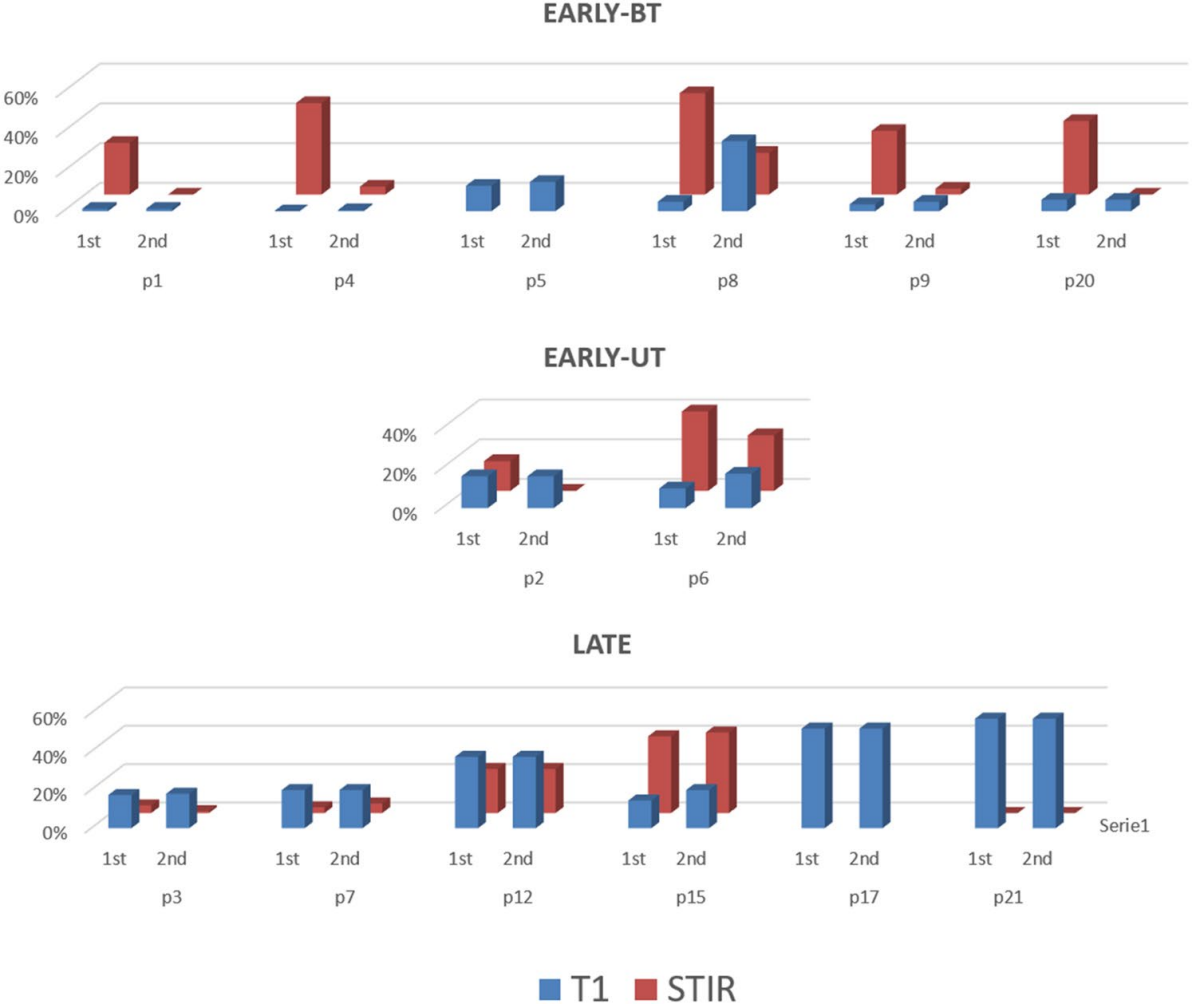
Longitudinal STIR%LB and T1-LB score variation. Variation of percentage of STIR positivity (red columns) and amount of fat replacement in T1 sequences (blue columns) between first and last MRI studies in LB in different patients divided in EARLY-BT, EARLY-UT, and LATE groups. STIR sequences represent a useful outcome measure for treatment response and disease remission in the EARLY groups. The best outcome (lower STIR and T1 at second MRI) is observed in patients treated early and aggressively (EARLY-BT group). Note the higher T1 score progression in cases of p8 (EARLY-BT) and p6 (EARLY-UT) who received IVIG later in disease course. T1 sequences do not show consistent progression in patients with longer disease duration (LATE group), sometimes resembling LGMD (p17, p21), while STIR sequences could reveal patients refractory to treatment or with disease relapses (p12, p15)

In this group of patients, the median STIR%LB score was 29% (range 0–51%, IQR 29.25) at first MRI and 2% (range 0–42%, IQR 21.5) at second MRI (*p* = 0.0024). This result was more evident in the 8 patients (8/14) of the whole EARLY group: STIR%LB was 37% (range 15–51%, IQR 24) at first MRI and 1% (range 0–40%, IQR 24.5) at second MRI (*p* = 0.0039), with a median time to the second MRI of 22 months (range 6–30, IQR 15.25). The average STIR variation between first and last MRI in LB (ΔSTIR-LB score) was −44% considering all patients. Nevertheless, considering subgroups, the average ΔSTIR-LB was −90.6% in EARLY-BT subgroup and −66.1% in EARLY-UT subgroup, while in the LATE group ΔSTIR-LB was + 21.3% (*p* < 0.0001) (Fig. 3).

A milder difference was also observed in T1 score change between first and last MRI study from a median score of 56.1 at first MRI (range 0–178) to 66.8 at second MRI (range 2–178). The ΔT1-LB was + 5.7% in EARLY-BT subgroup, compared to + 3.7% in EARLY-UT subgroup and + 6.1% in LATE group (Fig. 3).

**Fig. 4:**
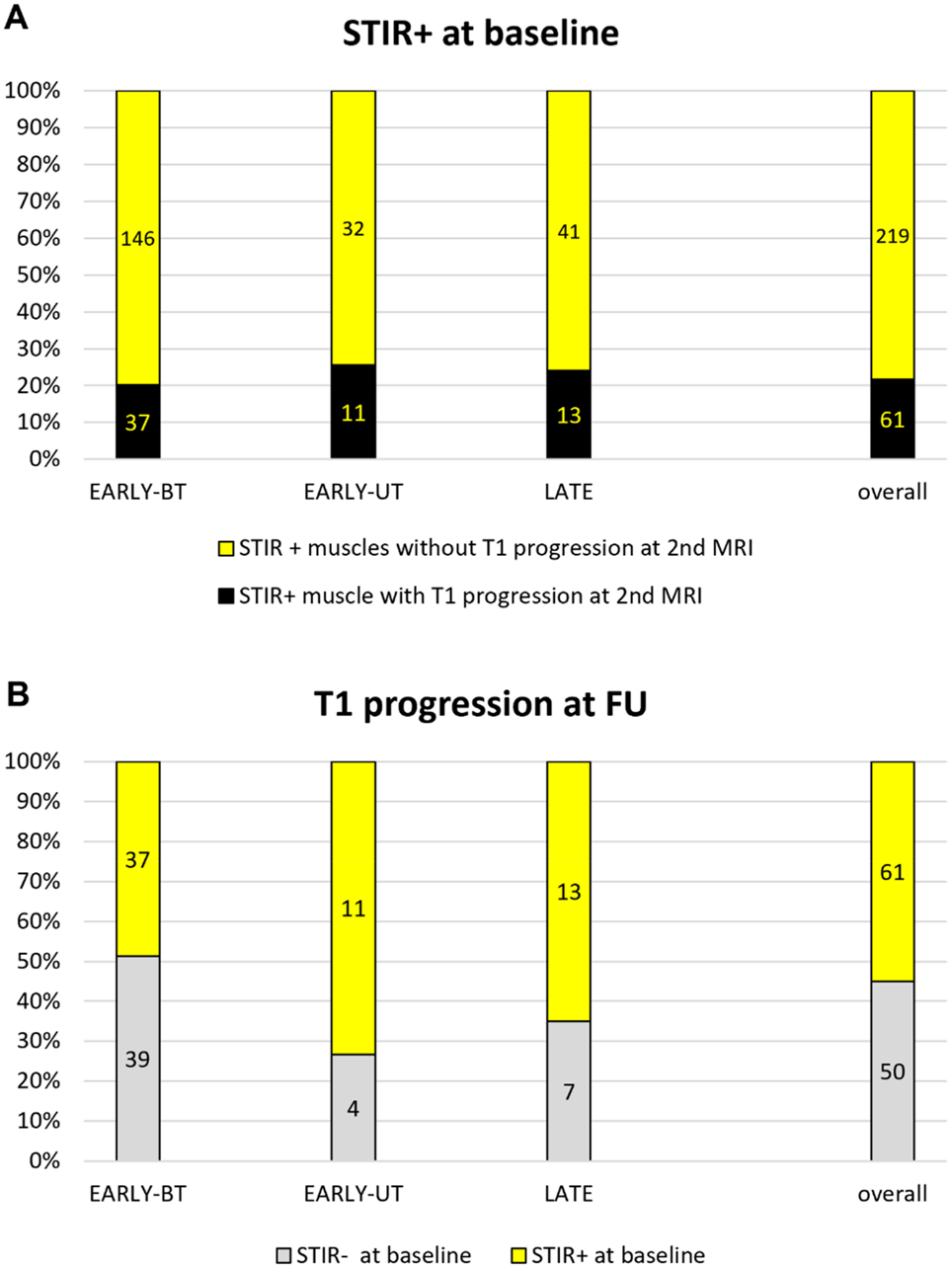
STIR positivity and T1 progression matching. Similar percentage among groups of T1 progression at FU in STIR positive muscles at baseline (**A**). Not all T1 progressed muscles at follow-up were STIR positive at baseline (**B**)

Overall, 22% of STIR positive muscles showed some degree of T1-score progression (at least 1 point) in the second MRI study (range 0–87%). The number of STIR positive muscles at first MRI that showed a progression of T1 score over the second MRI was similar among groups: 37/183 muscles in EARLY-BT subgroup (20.2%), 11/43 in EARLY-UT subgroup (25.6%) and 13/54 in LATE group (24.1%) (*p* = 0.6) (Fig. 4A). Conversely, the number of muscles that showed a T1 progression over the second MRI regardless of STIR positivity at first MRI was higher. In the EARLY-BT subgroup 39/76 muscles were not STIR-positive at first MRI (51.3%), 4/15 muscles in the EARLY-UT subgroup (26.6%) and 7/20 in the LATE group (35%) (*p* = 0.13) (Fig. 4B).

**Fig. 5:**
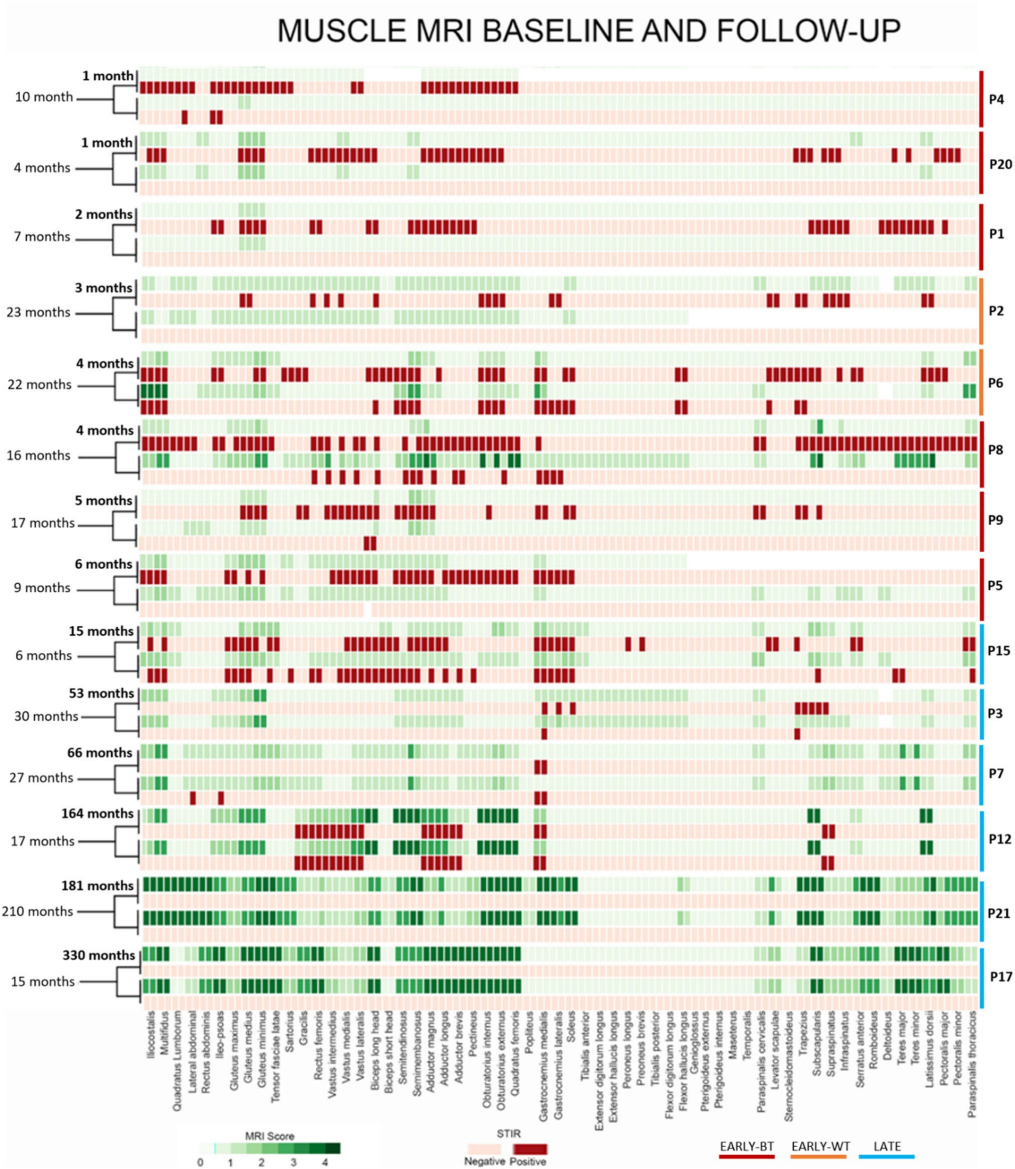
STIR and T1 matching heatmap at baseline and follow-up. Patients listed basing on timing at first MRI from disease onset in months (bold, left) and time elapsed between MRIs for each patient. Note the inverse gradient of STIR positivity decrease and T1 severity increase from up (EARLY groups) to down (LATE group), and the general overlap between STIR positive muscles (active phase of disease, EARLY group, up) and fat replaced muscles in T1 (most advanced phases of disease, LATE group, down). Each column represents one muscle of LB

The heatmap analysis shows a good overlap between STIR positive muscles and fat replaced muscles in T1 (Fig. 5).

### Correlation analysis

Inter-rater agreement (κ) for MRI scoring between observers was 0.87. In general, short disease duration correlated with higher STIR%LB score (*p* = 0.02, *R* = 0.5) while long disease duration correlated with higher T1-LB score (*p* = 0.02, *R* = 0.48).

Shorter timing of muscle MRI study from disease onset in untreated patients (EARLY-BT group) correlated with higher STIR%LB score at baseline and STIR change over time (ΔSTIR-LB) (*p* = 0.001, *R* = 0.67 and *p* < 0.001, *R* = 0.83 respectively), whereas MRI performed later in already treated patients (LATE group) correlated with higher T1-LB score (*p* = 0.004, *R* = 0.6).

Higher STIR%LB score correlated with higher change in T1 score at follow-up end (ΔT1 score) (*p* = 0.003, *R* = 0.76). Furthermore, lower changes in STIR signal at follow-up (ΔSTIR-LB) correlated with higher T1-LB score at follow-up end (*p* < 0.001, *R* = 0.85).

**Fig. 6:**
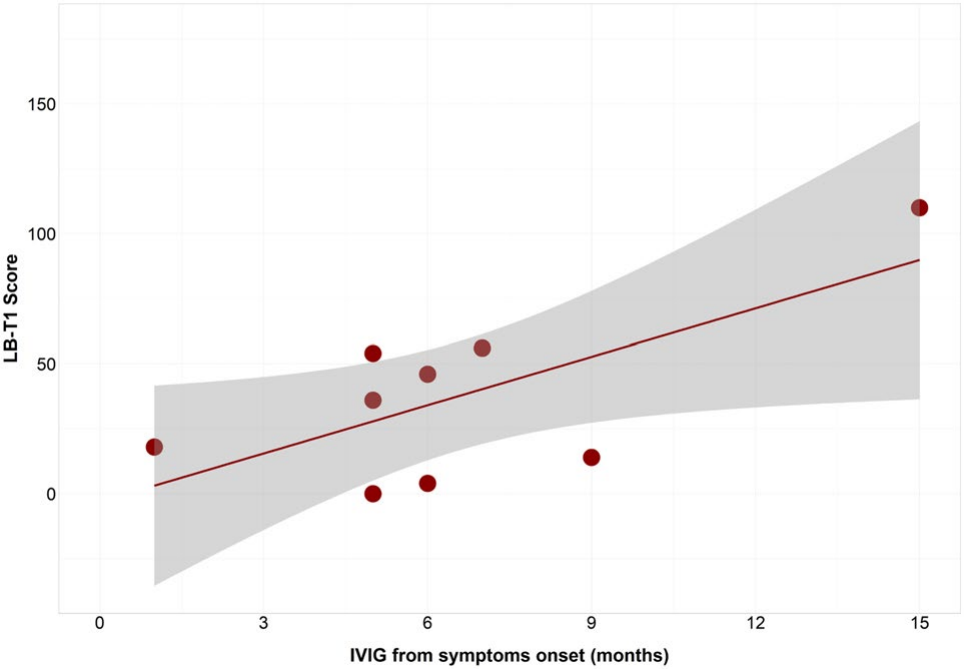
IVIG starting treatment and long-term fat replacement correlation. Correlation analysis shows that delayed treatment with IVIG (in months) is associated with higher fat replacement (LB-T1 score) at last FU (*p* = 0.03)

Both higher T1-LB and lower ΔSTIR-LB scores correlated with worse clinical outcome at follow-up end, specifically: with lower MRC-60 score (*p* < 0.001, *R* = 0.7 and *p* < 0.002, *R* = 0.6 respectively), lower disease remission rate (*p* = 0.001, *R* = 0.65 and *p* < 0.004, *R* = 0.56 respectively) and worst clinical outcome with higher diffuse/axial muscle weakness (*p* < 0.001, *R* = 0.73 and *p* = 0.001, *R* = 0.8 respectively). Noteworthily, the higher T1-LB score at follow-up end also correlated with late start of treatment (*p* = 0.03, *R* = 0.47) and in particular with delayed treatment with IVIG (*p* = 0.03, *R* = 0.7) (Fig. 6).

At baseline, CK values correlate with STIR%LB hyperintensity (*p* = 0.03, *R* = 0.266) and with its change during follow-up (ΔSTIR-LB) (*p* = 0.04, *R* =  − 0.6); CK also correlates with disease duration (*p* = 0.015, *R* =  − 0.223), and with T1-LB scores at baseline and at follow-up end (*p* = 0.009, *R* =  − 0.272) (*p* = 0.02, *R* =  − 0.16).

Variation of CK during follow-up correlated with ΔSTIR%LB hyperintensity (*p* = 0.04, *R* = 0.58).

Sex, age, and autoantibodies positivity were not associated with MRI alterations.

## Discussion

**Fig. 7:**
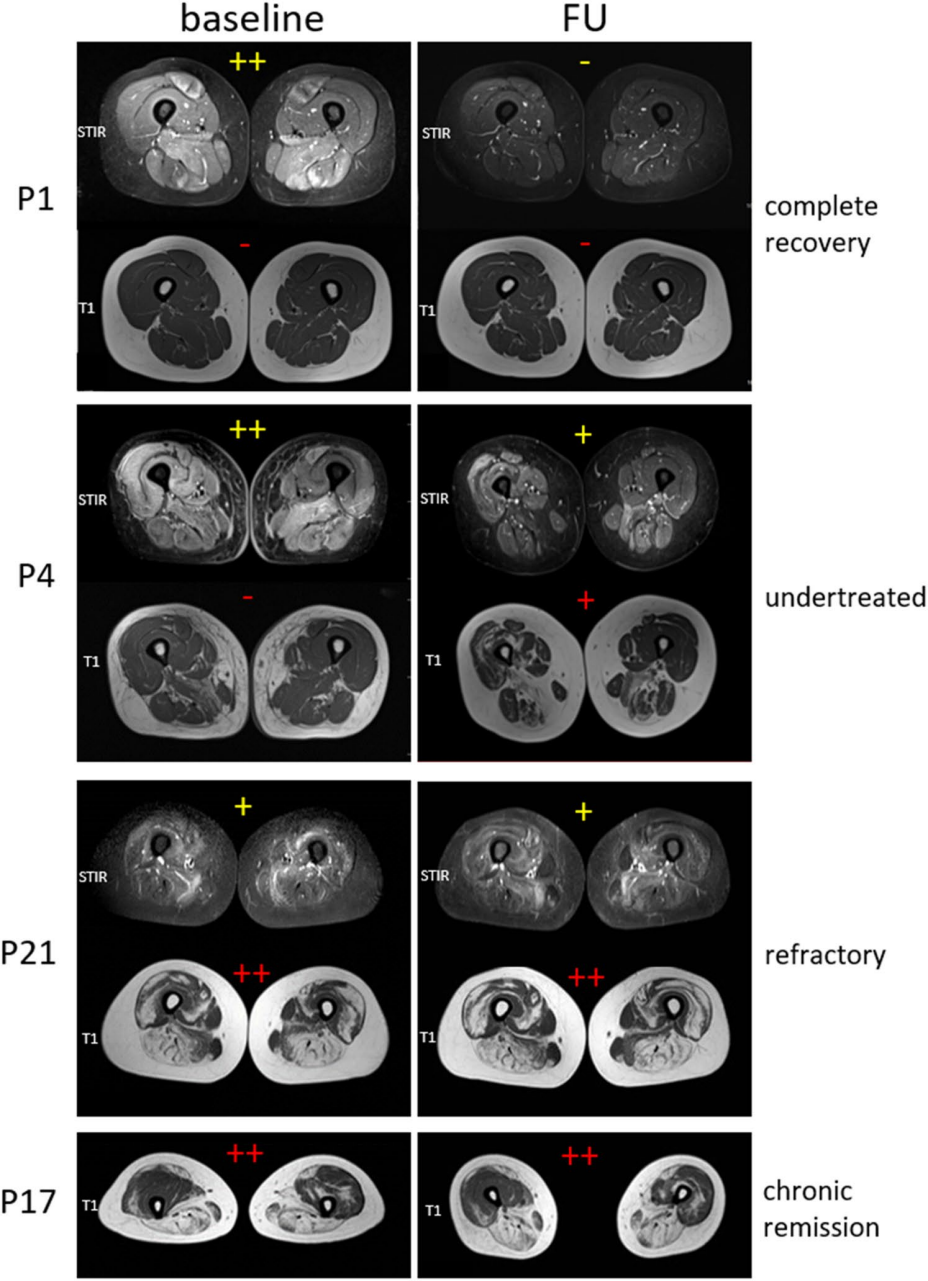
Muscle MRI findings. Representative cases of the importance of MRI study in the management of IMNM patients. Complete disease remission and clinical recovery with early aggressive immunotherapy (p1, EARLY-BT group). Incomplete disease remission (still positive STIR signal) and progression of fat replacement (T1 worsening) due to delayed start of IVIG (p4, EARLY-BT group). Persistence of STIR positivity and mild progression of fat replacement despite poly-immunotherapy in treatment refractory patient (p21, LATE group). STIR negativity (not shown) and T1 stationarity in a chronic-remission phase resembling LGMD (p17, LATE group)

Muscle MRI in IMNM is a sensitive biomarker for disease activity and treatment response when performed early in disease course, possibly before treatment start. As expected, our data show that STIR positive signal is higher in early stages of disease and in untreated patients and progressively decreases in treated patients and with long disease duration. In fact, ΔSTIR could represent a supportive outcome measure and early prognostic index for treatment response in IMNM, as it correlates with clinical disability and disease remission other than MRC-60 score and CK variation during follow-up. Even if MRC significantly varied during follow-up, we introduced an overall clinical evaluation to better understand retrospectively the clinical course of our cohort of patients (Table 1). Indeed, lower variation of ΔSTIR correlates to higher degree of fat replacement (T1-score) at follow-up—corresponding to higher disability—as it occurs in undertreated patients or refractory conditions. T1 sequences provides information about the overall burden of disease, representing the most important outcome measure which positively correlates with muscular weakness and clinical impairment. Higher T1-score at follow-up end is also associated with delayed start of treatment from disease onset, in particular with delayed start of IVIG therapy, confirming its key role in inducing disease remission in IMNM. The importance of IVIG in the management of IMNM is represented by clinical history of three representative patients belonging to the EARLY-BT group: P1, P4 (Fig. 7) and P3. P1 represents the classical management of IMNM characterized by a progressive adding-on therapy, started with prednisone as first-line therapy with partial improvement, subsequent add-on of immunosuppressive agents for second-line therapy and lastly, the use of IVIG at 6 months from disease onset to reach a disease remission and complete clinical recovery. Conversely P4, despite having a similar history with early start of first and second-line treatments, started IVIG therapy only 15 months later disease onset with marked improvement, but considerable residual disability due to the fat replacement occurred in muscles, confirmed by marked increase of T1 score (ΔT1) at follow-up MRI study. Finally, P3 obtained disease control only by IVIG as first-line therapy because of contraindication of corticosteroids and immunosuppressive agents, supporting the beneficial use of IVIG also as first-line therapy alone.

On the other hand, T1-score is higher in LATE group. Most of these patients are in a remission phase of the disease and muscle MRI provides information on the consequences of past periods of muscle inflammation (at onset and during relapses) and correlates with clinical disability. Longer disease course is generally associated to higher degree of fat replacement, probably because of possible periods of low disease control (due to treatment refractory), or even because of an initial period of misdiagnosis (e.g. seronegative IMNM for toxic myopathies). In these cases, especially if there is a permanent disability at neurological examination, persistence of STIR positive signal can help to assess treatment unresponsiveness or even detect disease relapses. Conversely STIR negativity is associated to chronic or remission phases. In most chronic cases, muscle MRI could resemble that of inherited myopathies or muscular dystrophies. Two cases of LATE group are representative: P21 and P17 (Fig. 7) that performed first MRI very late in disease course. In the former case, MRIs were performed during a lasting period of disease activity and refractory to different immunosuppressive treatments, while in the latter MRIs were both performed during a period of disease remission. In these cases, STIR negativity helps to identify disease remission despite residual muscular weakness from those with STIR positivity due to still active disease, which could improve with alternative or more aggressive immunotherapy, underling the importance of MRI to assess disease remission regardless of residual clinical disability.

In general, we observed a good overlap between inflammation and fat replacement (Fig. 5), in accordance with the assumption that fat replacement in inflammatory myopathies is the consequence of muscle inflammation. In our cohort, during the period of longitudinal evaluation, about 20% of STIR positive muscles progressed in T1 score at follow-up, regardless of belonging group, while more than 50% of muscles that showed T1-score progression were not STIR positive in the first MRI. This unexpected finding could have different explication. First, STIR sequences have not enough sensitivity to detect all inflammatory processes in muscles, in fact muscle biopsies in patients affected by IIMs can show inflammatory infiltrates also in STIR negative muscles, suggesting that cellular mechanisms of inflammation are not constantly captured by muscle MRI, probably due to lack of muscle oedema above the threshold needed for MRI detection. Nevertheless, inflammatory infiltrates still ensure the pathophysiological mechanism underlying the muscle damage and fat replacement progression. On the other hand, MRI can detect muscle inflammation in patients with no clinical sign of muscular involvement and in biopsy-negative patients [27, 28] because of sampling errors, or conversely because of STIR hyperintensity could occur for a transitional period. Probably, other mechanisms than muscle inflammation could participate to the pathophysiology underlying fat replacement and disease progression in inflammatory myopathies.

Even if a preferential pattern of involvement could be recognized in our cohort of patients according to those already described [15, 29], no significant differences among different serotypes have been observed. The only minor variability concerned the pattern of muscle STIR positivity—which was more prominent in lumbar, *gluteus minimus* and hamstring muscles in SRP, *gluteus medius* and pelvic muscles in HMGCR and quadriceps and hamstring muscles in seronegative patients suggesting a possible implication of the antibody-related disease mechanism in the muscle-specific susceptibility, a well-known phenomenon frequently observed in inherited myopathies [30]. Certain muscles are early involved in disease course, while others remain spared until the late-end stage of disease, even if all muscles in the body share the same genetic background. This variability represents the cornerstone of the disease-specific patterns of muscle involvement described in several myopathies and muscular dystrophies. Mechanisms underlying the variable muscle-specific susceptibility/resistance to injury is not yet fully understood but it is probably the result of complex interactions between different gene expression and modulation between muscles, and muscle tissue environment. Different inflammatory pathways among IIMs could lead to the preferential involvement of certain muscles as inclusion body myositis (IBM) (*flexor digitorum profundus*, *quadriceps* and *gastrocnemius medialis*), DM and Antisynthetase syndrome (ASS) (symmetrical pelvic and relative adductor-sparing thigh muscles) or myasthenia-myositis association (upper limb extensors and more prominent in myotendinous junctions) [19, 31–33], helping to differentiate the pattern of muscle involvement from IMNM. The predominant axial and pelvi-femoral muscle involvement with prominent STIR positivity could also help to differentiate IMNM from other myopathies [34–39]. IMNM may resemble limb-girdle muscular dystrophies (LGMD) not only in HMGCR serogroup [40], but also in SRP or seronegative patients, as in the cases P17, P20 and P21 of the LATE group. The risk to develop severe clinical phenotypes resembling LGMD should be linked to history of long disease duration in misdiagnosed, undertreated or treatment-refractory patients, rather than to a specific serological group.

This study has some limitations. The major is due to the retrospective analysis and the heterogeneity of the sample, with MRIs performed at different time from diagnosis. The small size of our cohort did not allow us to have a relevant number of patients in each serological group possibly reducing the statistical differences between them. MRI study did not include upper body for all patients, and during follow-up it was performed for clinical purposes only (not following a standardized protocol). Finally, we could not correlate the MRI results with muscle biopsy, because they were not performed concurrently in all cases.

## Conclusions

Muscle MRI is a sensitive biomarker for monitoring disease activity and therapy response in IMNM, notably if performed early in disease course and before treatment start, and could represent a supportive outcome measure and early prognostic index. In particular higher STIR positivity at baseline and delayed treatment start (in particular with IVIG) are associated to higher degree of fat replacement during disease course and worst clinical outcome.
